# Prevalence of suicide in adolescents and youth at Ultra High Risk for Psychosis - A Systematic Review and Meta-Analysis

**DOI:** 10.1192/j.eurpsy.2025.340

**Published:** 2025-08-26

**Authors:** S. H. Ang, S. Venkateswaran, K. N. C. Naidu, M. Bakulkumar Goda, G. Kudva Kundadak

**Affiliations:** 1 Yong Loo Lin School of Medicine, National University of Singapore; 2 Department of Psychological Medicine, National University Hospital; 3 Saw Swee Hock School of Public Health, National University of Singapore; 4 Department of Psychological Medicine, Yong Loo Lin School of Medicine, National University of Singapore; 5 Department of Psychological Medicine, Alexandra Hospital, Singapore, Singapore

## Abstract

**Introduction:**

Suicide remains a major risk factor for individuals suffering from Schizophrenia. Recent studies have established that patients in the prodromal Schizophrenia state (i.e. Ultra High Risk for Psychosis) also experience significant rates of suicidal ideation and behaviour. However, less is known about the prevalence of suicidality among the adolescent and youth UHR population, a demographic particularly vulnerable to the psychosocial and environmental risk factors of psychosis.

**Objectives:**

This review aims to synthesise the existing literature on the prevalence of suicidal ideation and behaviour in the adolescent and youth at Ultra High Risk for Psychosis (UHR), and the relevant associations between suicidality and its correlates.

**Methods:**

The databases PsycINFO, PubMed, Embase, Cochrane Library, Web of Science and Scopus were accessed up to July 2024. References within selected journals were further hand-searched for eligible articles. Keywords and controlled vocabulary used consisted of: (‘Ultra High Risk’ OR ‘At Risk Mental State’ OR ‘Clinical High Risk’) AND (‘Schizophrenia’ OR ‘Psychosis’) AND (‘Self-Harm’ OR ‘Suicide’ OR ‘NSSI’) AND (‘Adolescent’ OR ‘Youth’). Articles that included participants with an established diagnosis of schizophrenia or intellectual disability, history of frank psychotic episodes and extended use of antipsychotics were excluded. A meta-analysis of prevalence was subsequently performed for lifetime suicidal ideation, lifetime non-suicidal self-injury, lifetime suicidal attempt and current suicidal ideation. A narrative review was also carried out for the correlates of suicidality amongst the adolescent and youth UHR population.

**Results:**

Fourteen studies were included in this meta-analysis. Meta-analysis revealed a high prevalence of lifetime suicidal ideation (56%), lifetime non-suicidal self-injury (37%), lifetime suicidal attempt (25%) and current (2-week) suicidal ideation (58%). Narrative review revealed that personal transition to psychosis and positive family history of psychosis was strongly associated with suicidal attempt, while CAARMS (Comprehensive Assesment of At Risk Mental States) severity score was associated with suicidal ideation. Depression was strongly associated with both suicidal attempt and suicidal ideation, whereas positive symptoms and self-disturbance were associated with self-injury.

**Conclusions:**

The prevalence of suicidal ideation and behaviour among UHR adolescent and youth is high and comparable to the general UHR population. Effective detection and management of suicide risk will be especially crucial in the adolescent UHR population. Existing measures that mitigate suicide risk in the general UHR population should be adopted for the youth and adolescent context.

**Disclosure of Interest:**

None Declared

